# High Resolution CT Imaging Dynamic Follow-Up Study of Novel Coronavirus Pneumonia

**DOI:** 10.3389/fmed.2020.00168

**Published:** 2020-05-04

**Authors:** Xuefang Lu, Wei Gong, Zhoufeng Peng, Feifei Zeng, Fang Liu

**Affiliations:** Department of Radiology, Renmin Hospital of Wuhan University, Wuhan, China

**Keywords:** COVID-19, pneumonia, tomography, X-ray computed, CT imaging

## Abstract

**Objective:** To explore the clinical characteristics and dynamic follow-up changes of high resolution CT (HRCT) in 270 patients with Coronavirus Disease 2019 (COVID-19) pneumonia.

**Methods:** Two hundred seventy COVID-19 pneumonia patients were retrospectively analyzed, including 146 males and 124 females, with median age of 51 (9,89). The clinical features, laboratory examination indexes and HRCT evolution findings of 270 COVID-19 pneumonia patients were analyzed.

**Results:** 264 cases (95.74%) were positive at the first time nucleic acid test, 6 cases (2.22%) were negative, after multiple inspections, 270 cases (100%) were positive. According to the number of lung segments involved in the lesion, the lesions range from <30% of the lung area (Common type), 30–50% (Severe type), and> 50% (Critical type). At the first CT exam, 136 cases (50.37%) of the common type, 89 cases (32.96%) of the severe type and 45 cases (16.67%) of the critical type. At the second CT exam, 84 cases (31.11%) of the common type, 103 cases (38.15%) of the severe type and 83 cases (30.74%) of the critical type. In the third CT exam, there were 151 cases (55.93%) of the common type, 86 cases (31.85%) of the severe type and 33 cases (12.22%) of the critical type. The differences in image typing were statistically significant (*P* < 0.05). During this study, a total of 173 patients (64.08%) were recovered after treatment.

**Conclusion:** In some epidemiological backgrounds, CT imaging manifestations and evolutionary characteristics are of great significance for early warning of lung injury, assessment of disease severity, and assistance in clinical typing and post-treatment follow-up.

Since the first report of unexplained viral pneumonia in Wuhan, Hubei Province, China, on December 8, 2019, with the advent of pathogenic detection methods, a large number of cases have been confirmed in various provinces across the country. As of February 14, 2020, China 63,918 cases were confirmed (1), and such cases were successively confirmed abroad. On January 12, 2020, the World Health Organization named the novel coronavirus “2019 novel coronavirus (2019-nCoV).” On February 11, 2020, the International Virus Classification Commission the virus research team named the novel coronavirus “SARS-CoV-2,” and the same day the World Health Organization named the disease caused by the novel coronavirus “Coronavirus Disease 2019, COVID-19” (2), the pneumonia caused by it, called COVID-19 pneumonia.

COVID-19 pneumonia patients usually have flu-like symptoms clinically. Unlike common influenza virus infections, SARS-CoV-2 is highly pneumophilic and can easily cause viral pneumonia. It has the characteristics of rapid disease development and high infectivity (3). A few patients may develop hypoxemia, acute lung injury, and severe patients may develop severe pneumonia, such as acute respiratory distress syndrome and respiratory failure, which may even lead to death (3, 4). The clinical characteristics and high resolution CT (HRCT) imaging data of 141 patients with COVID-19 diagnosed by Renmin Hospital of Wuhan University from January 20, 2020 to February 21, 2020 were retrospectively analyzed. The aim is to explore the clinical characteristics and dynamic follow-up changes of HRCT in patients with COVID-19 pneumonia.

## Materials and Methods

### General Information

**Figure 1:**
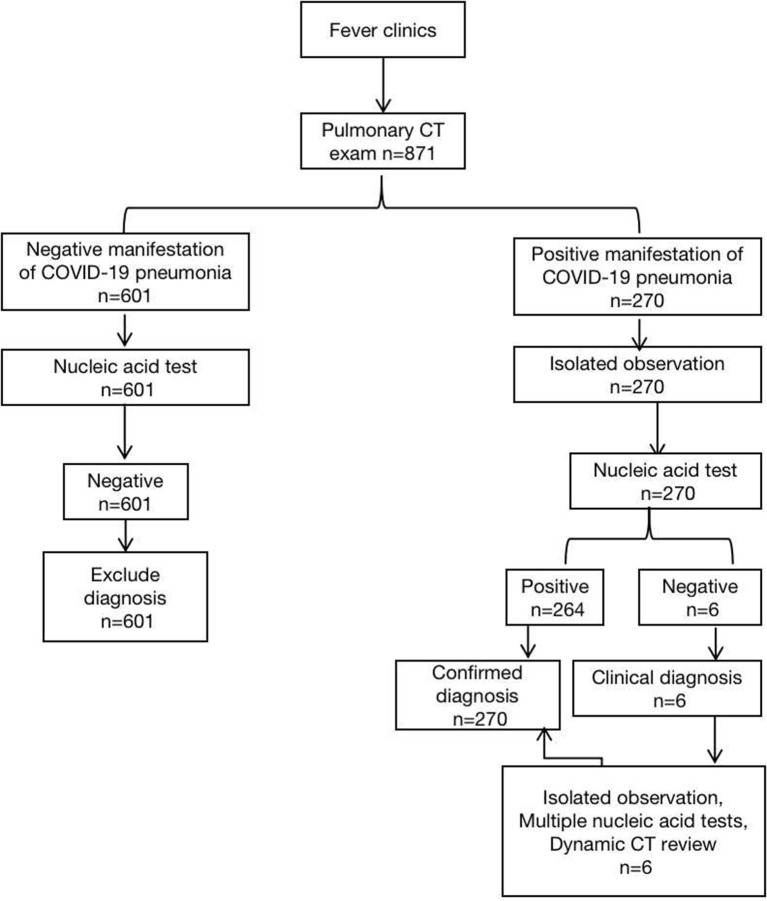
Flow chart of CT examination of fever clinics in Hubei Province.

A retrospective analysis of 871 patients who underwent parallel HRCT scans of the Renmin Hospital of Wuhan University from January 20 to February 21, 2020 due to fever. The patient triage process was in accordance with the National Health and Health Committee of the People's Republic of China, “Pneumonia diagnosis and treatment plan (trial version 5)” (Figure 1), confirmed the inclusion criteria of new coronavirus pneumonia: (1) all patients with throat swabs were lysed and extracted by nucleic acid kit to calculate the fluorescence RT- The viral nucleic acid gene sequence was detected by PCR and compared with the 2019-nCoV nucleocapsid protein gene (nCoV-NP) and the 2019nCoV open reading coding frame lab (nCoVORFlab) sequence. The final result was positive (4); (2) The quality of HRCT imaging in chest are qualified without obvious artifacts and missing images. A total of 270 COVID-19 pneumonia patients were included, 146 males and 124 females, with a median age of 51(9,89) years and no pregnant women, of which 2 (0.74%) was <12 years old, and 38(14.07%)were 12–24 years old, 65 cases (24.07%) between 25 and 44 years old, 103 cases (38.15%) between 45 and 64 years old, 59 cases (21.85%) between 65 and 84 years old, and 3 cases (1.11%) over 85 years old. 84 (31.11%) of the 270 patients with COVID-19 pneumonia had underlying diseases, including 26 cases(9.63%) of diabetes, 29 cases(10.74%) of hypertension, 28 cases(10.37%) of atherosclerosis and coronary heart disease, and some patients were at the same time suffering from many of the above chronic underlying diseases. All 270 patients with COVID-19 pneumonia were included in the laboratory examination (peripheral white blood cell count, lymphocyte ratio). This study was approved by the Ethics Committee of Renmin Hospital of Wuhan University, and informed consent was dispensed with.

### Research Methods

Chest HRCT was performed using 64 multidetector CT (Optima 680,GE Healthcare) and 16 multidetector CT (Brightspeed, GE Healthcare). Scanning parameters is as follows: tube voltage 120 kV, tube current 200 mA, layer spacing 5 mm, acquisition layer thickness 0.625 mm, scanning time <5 s. The standard lung window is set to 530–430 HU, the window width is 1 400–1 600 HU; the mediastinal window is set to 35–40 HU, the window width is 300–350 HU. The scanning range was from the thorax entrance to the posterior costal angle. The HRCT raw data were reconstructed using bone algorithm.

Two radiologists with extensive work experience performed a visual evaluation of the HRCT images and recorded image performance, lesion distribution and extent, lung injury index, image features and other accompanying image signs. In the lung window image, the distribution of the lesions and the main imaging features (ground glass opacity < GGO>, focal exudative plaque shadows, and large consolidation shadows) were recorded. According to the distribution of the lung segments, the two lungs are approximately equal to 20 parts (of which the S1+2,S3,S7+8 segment of the left lung should be regarded as 2 equal parts, respectively. For each lung segment involved, the main imaging feature of the lesion is estimated to be 5%. According to the number of lung segments involved in the lesion, the lesions range from <30% of the lung area (Common type), 30% to 50% (Severe type), and> 50% (Critical type) (5). When the diagnosis results of the two doctors are inconsistent, the third senior doctor is introduced for arbitration to reach an agreement.

### Statistical Methods

All data were analyzed using SPSS 22.0 statistical software. Age is non-normally distributed measurement data, expressed as the median (upper, lower quartiles); examination time is measured data that conforms to the normal distribution, expressed as (x ± s); clinical characteristics and imaging features data were expressed by frequency and rate. Differences in different CT typing rates were performed by chi-square test, *P* < 0.05 was considered statistically significant.

## Result

### Clinical Features at Preliminary Diagnosis

Of the 270 patients with COVID-19 pneumonia, 262 (97.04%) had fever (> 37.5°C), 200 (74.07%) had cough, 21 (7.78%) had headache, 51 (18.89%) had expectoration, and 139 cases (51.48%) had breathless. Eight cases (2.96%) with symptoms other than respiratory tract, such as diarrhea. Laboratory tests of 270 COVID-19 pneumonia patients showed white blood cell count decreased in 72 cases (26.67%) and lymphocyte ratio decreased in 134 cases (49.63%) (Table 1).

**Table 1:** General information.

**Item**	**Demographic**		**Clinical manifestations**						**Laboratory examination**		
	**Male**	**Female**	**Fever**	**Cough**	**Headache**	**Expectoration**	**Breathless**	**Diarrhea**	**PCR(+)**	**WBC↓**	**LYMPH%↓**
Cases	146	124	262	200	21	51	139	8	270	72	134
Incidence (%)	54.07	45.93	97.04	74.07	7.78	18.89	51.48	2.96	100	26.67	49.63

*The median age of all subjects is 49 years (9, 87), 2 cases <12 years (0.74%), 38 cases (14.07%) 12–24 years, 65 cases (24.07%) 25–44 years, One hundred and three cases (38.15%) 45– 64 years, 59 cases (21.85%) 65–84 years, and 3 cases (1.11%) >85 years*.

Sixty two (22.96%) patients of COVID-19 with bacterial infections at the same time, of which 41 (15.19%) had Streptococcus pneumoniae infection, 16 (5.93%) had *Mycobacterium tuberculosis, Haemophilus influenzae* or *Staphylococcus aureus*, 5 (1.85%) with other bacteria infection.

### Chest HRCT Imaging Features

All patients between symptoms started and went to hospital with CT exam <1 week. Two hundred seventy COVID-19 pneumonia patients had abnormal HRCT images of chest at first diagnosis. Sixty-four cases (23.70%) showed unilateral pulmonary lobe lesions, mainly subpleural distribution; 206 cases (76.30%) had bilateral lung lobe involvement. All 270 patients with COVID-19 pneumonia had different levels of intrapulmonary lesions, of which 68 (25.19%) patients were involved in all lung lobes and segments of the lungs; in localized cases, the right lower lobe was most affected, with 40 cases (14.81%). Among the 270 patients, 187 cases (69.26%) of right lung lesions have a wider range than left lung lesions, and 83 cases (30.74%) of left lung lesions have a wider range than right lung lesions; the lower lobe lesions are more than the upper lobe lesions. Eighty-six cases (61.11%) had a wide range of lesions, 50 cases (18.52%) had a larger range of lesions in the upper lobe than in the lower part, and 55 cases (20.37%) had roughly equivalent lesion range in the upper and lower parts.

One or more of the following signs were seen in 270 patients with COVID-19 pneumonia. The HRCT images of the chest of 99 patients(36.67%) showed ground glass opacity(GGO), mainly under the pleura; 44 cases(16.30%) showed GGO with focal consolidation; 52 cases (19.26%) of small patch edge blur density increased; 38 cases (14.07%) of large patch consolidation; 92 cases (34.07%) bronchial vascular bundle thickening and vascular crossing signs; 9 cases (3.33%) had air bronchial signs (**Figure 4**); 13 cases (4.81%) had solid nodules with a diameter of <0.5 cm in the same lung lobe; 10 cases (3.70%) of grid shadows or stripe shadows; 43 cases (15.93%) of diffuse lung lobe lesions, showing “white lung.”

Of the 270 COVID-19 pneumonia patients in this group, 69 (25.56%) had chronic respiratory diseases (chronic bronchitis, bronchiectasis, emphysema, bullae), and 86 (31.85%) showed signs of cardiovascular disease (valve calcification, aortic wall calcification, coronary arterial wall calcification), 13 patients (4.81%) had bilateral pleural effusion, and 8 patients (2.96%) had mediastinal or bilateral hilar lymphadenopathy (short diameter ≥ 1.0 cm), 11 cases (4.07%) were accompanied by abnormal signs of the upper abdomen, including fatty liver, liver cysts, and gallbladder stones.

In the particular epidemiological environment, patients suspected to be COVID-19 in this area need to undergo CT examination before performing nucleic acid test. Patients with SARS-CoV-2 negative molecular detection test were observed, and none of them had typical CT manifestations of COVID-19 pneumonia, so they were not included in this study (Figure 1).

### CT Classification of Lung Lesions, Changes in Treatment Outcomes

**Figure 2:**
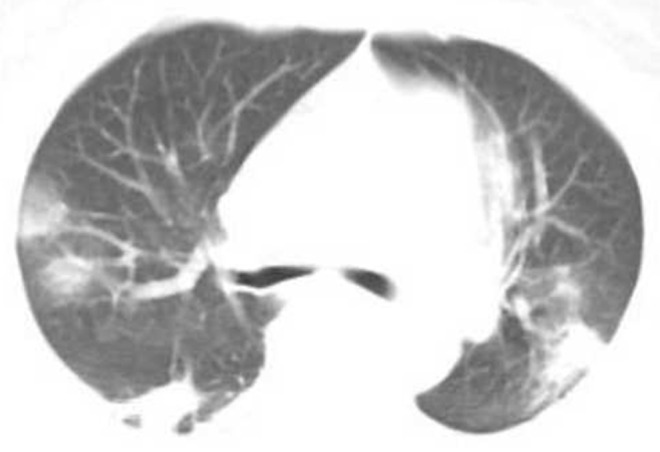
Female, 56 years, with fever pending investigation. HRCT plain scan axial lung window showing lesions in bilateral lung lobes at the first time; This figure shows common type lesions.

**Figure 3:**
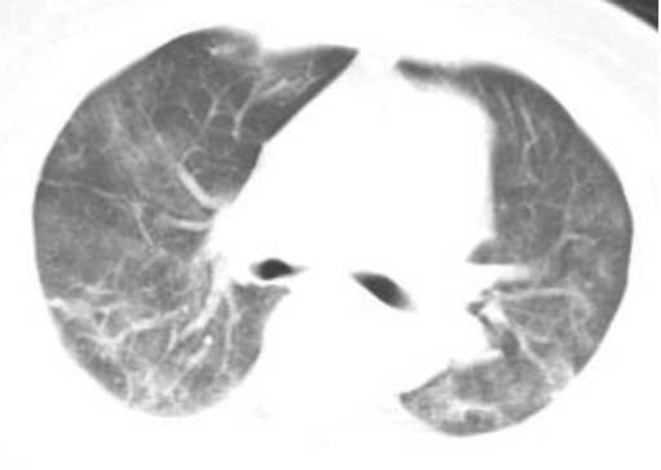
Female, 56 years, with fever pending investigation. HRCT plain scan axial lung window showing lesions in bilateral lung lobes at the first time. The same patient was re-examined after 4 days, and the lesion range increased, showing the severe type lesion.

**Figure 4:**
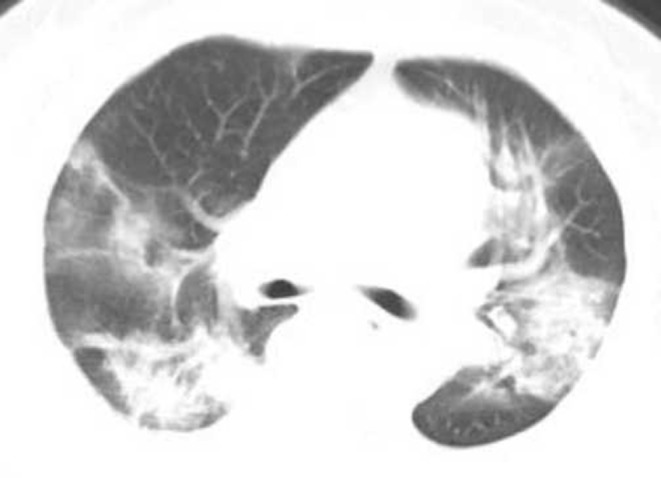
Female, 56 years, with fever pending investigation. HRCT plain scan axial lung window showing lesions in bilateral lung lobes at the first time. The same patient was re-examined after 10 days, and the lesion range was further enlarged, showing the critical type lesion.

**Figure 5:**
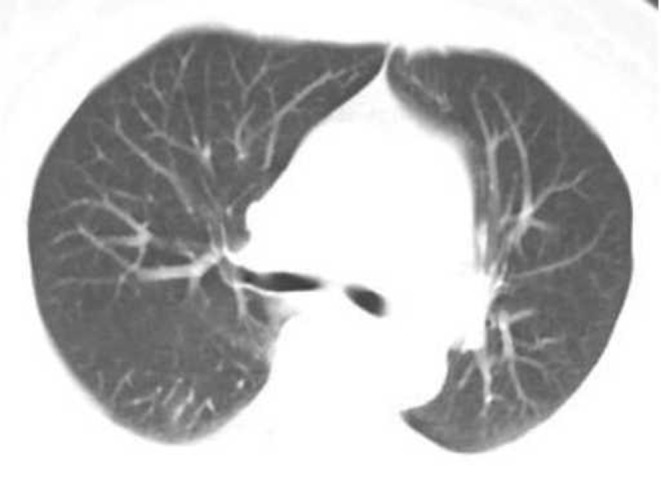
Female, 56 years, with fever pending investigation. HRCT plain scan axial lung window showing lesions in bilateral lung lobes at the first time. The same patient was re-examined after 15 days, the patient was recovered after treatment, no lesions on her CT imaging.

Two hundred seventy COVID-19 patients with at least 4 chest HRCT tests. The average interval time is shown in Table 2. At the first CT exam, 136 cases (50.37%) of the common type (Figure 2), 89 cases (32.96%) of the severe type and 45 cases (16.67%) of the critical type. At the second CT exam, the range of lesions decreased in HRCT imaging of some patients, 84 cases (31.11%) of the common type at this time, and the range of lesions increased in HRCT imaging of other patients, 103 cases (38.15%) of the severe type and 83 cases (30.74%) of the critical type at the same period (Figure 3). In the third CT exam, the range of lesions decreased in most of patients' HRCT imaging, there were 151 cases (55.93%) of the common type, 86 cases (31.85%) of the severe type and 33 cases (12.22%) of the critical type (Figure 4). The differences in image typing were statistically significant (*P* < 0.05) (Table 2). Among the 136 cases of the common type at the first CT exam, 102 patients (37.78%) were recovered (Figure 5), 29 patients (10.74%) always showed common type, and 5 cases (1.85%) of the critical type after treatment. After treatment, among the 89 cases of the severe type at the first CT exam, 45 patients (16.67%) were recovered, 36 patients (13.33%) always showed severe type, and 8 cases (2.96%) of the critical type. Among the 45 cases of the critical type at the first CT exam, 26 patients (9.63%) were recovered, 10 patients (10.74%) always showed critical type, otherwise the range of lesions increased in 9 patients (3.33%) after treatment. In this study, a total of 173 patients (64.08%) were recovered (Figure 5), 75 patients (27.78%) always showed the same type like before, and 22 patients (8.15%) showed the critical type lesions at last time until this manuscript written. Unfortunately, 9 patients (3.33%) death after active treatment. (specific time changes are shown in Table 2).

**Table 2:** Evolution of lung lesions CT typing.

	**First CT exam**		**Second CT exam**		**Third CT exam**		**Outcome**				**CT typing difference**
	**Average time (d)**	**Cases (%)**	**Average time (d)**	**Cases (%)**	**Average time (d)**	**Cases (%)**	**Average time (d)**	**Cure cases (%)**	**Maintain cases (%)**	**Deterioration cases (%)**	
Common	2.61 ± 1.41	136/270 (50.37)	3.34 ± 1.17	84/270 (31.11)	5.59 ± 1.76	151/270 (55.93)	16.25 ± 1.24	102/270 (37.78)	29/270 (10.74)	5/270 (1.85)	*p* < 0.05
Severe	5.31 ± 2.30	89/270 (32.96)	4.01 ± 1.78	103/270 (38.15)	7.32 ± 1.43	86/270 (31.85)	19.48 ± 1.65	45/270 (16.67)	36/270 (13.33)	8/270 (2.96)	*p* < 0.05
Critical	5.91 ± 1.81	45/270 (16.67)	4.22 ± 1.46	83/270 (30.74)	7.56 ± 1.76	33/270 (12.22)	24.96 ± 1.82	26/270 (9.63)	10/270 (3.70)	9/270 (3.33)	*p* < 0.05

## Discussion

### Clinical Characteristics of Preliminary Diagnosis

The novel coronavirus and the severe acute respiratory syndrome coronavirus (SARS-CoV) (6), the Middle East Respiratory Syndrome coronavirus (MERS-CoV) (7) also belong to the Coronaviridae-specific non-segmented RNA virus (8), and the novel coronavirus has a high mutation rate (9), has highly toxic, and easier to spread from person to person (10). COVID-19 pneumonia is common in adults and rare in children (11). Only one patient under the age of 12 was found in this study. The ratio of leukocytes and lymphocytes in patients enrolled in this study decreased, suggesting that patients with multiple organ functions may be damaged once they are infected with SARS-CoV-2 virus (12). In this study, 262 (97.04%) COVID-19 pneumonia patients were first diagnosed with fever symptoms, showing that this symptom is of great significance in the clinical pre-test triage of this disease (13), but it should be pointed out that all patients in this group were feverish. At the clinic, there may be a shift. In addition, a small number of patients may have diarrhea and other respiratory manifestations (14). The lack of obvious specific clinical manifestations increases the difficulty of diagnosis, easily leads to missed diagnosis or misdiagnosis, and increases the chance of infection. Under the epidemiological conditions, the medical staff receiving the first consultation must be vigilant and perform chest HRCT screening and virological testing in a timely manner.

### Chest HRCT Imaging Features

All patients between symptoms started and went to hospital with CT exam <1 week, but we don't know when they first got the virus. The medical preprint platform medRxiv published a new study on the incubation period of the new coronavirus disease (COVID-19) in collaboration with the Peking University team and the National Institutes of Health (NIH), entitled “Estimation of incubation period distribution of COVID-19 using disease onset forward time: a novel cross-sectional and forward follow-up study.” The research team pointed out that using the well-known renewal theory in probability to estimate, it was found that the incubation period of new crown disease may be longer than known, about 10% of patients have an incubation period longer than 14 days. This also means that approximately 10% of COVID-19 patients will not experience symptoms until 14 days after infection. In general, the incubation period of an infectious disease refers to the time between the infection of the patient and the appearance of the first symptoms. An accurate understanding of the incubation period will help to provide the best length of isolation for disease control and play a role in studying the mechanism of disease transmission and development (15).

Two hundred seventy patients with COVID-19 pneumonia had intrapulmonary lesions of different locations and scopes. Among the localized lesions, 40 (14.81%) patients had localized lesions mainly involving the right lower lobe, which may be related to SARS-CoV-2 target cells may be located in the lower respiratory tract (6, 16). Because patients with COVID-19 pneumonia can have bacterial infections at the same time, and may be affected by the host's immune status and the potential pathophysiology of viral pathogens (17), the HRCT manifestations of COVID-19 pneumonia patients are diverse. The HRCT of 270 COVID-19 pneumonia patients all showed varying degrees of lung lesions. This was related to SARS-CoV-2 acute respiratory disease infection to a certain extent, which could lead to respiratory epithelial and airway mucosal damage. It is the adhesion and proliferation of pathogenic microorganisms. creating certain conditions, patients can progress to pneumonia or even respiratory failure (17). In this study, the first chest CT of 43 patients (15.93%) showed “white lung,” suggesting that the inflammatory lung disease progressed rapidly to the severe stage (18).

In this study, 99 patients (36.67%) with GGO in chest CT imaging. These signs are mainly seen in patients with early disease and mild symptoms of COVID-19 pneumonia, which may be due to infection resulting in alveolar swelling, small exudation of the alveolar cavity, and inflammation of the alveolar space, caused increased lung density (18). In this study, 44 cases (16.30%) presented ground-glass shadows with focal consolidation or multiple irregular consolidation areas along the bronchial vascular bundle and diffuse GGO, and 52 cases (19.26%) showed patchy blurring. Thirty-eight cases (14.07%) showed large-scale consolidation images. These different image manifestations are mainly due to the inflammatory changes in the parenchyma of the lung, which can change according to the progress of the disease course or the treatment of the disease. Signs mainly appear in patients with COVID-19 pneumonia whose disease is in progress. Ninety-two patients (34.07%) showed thickened bronchial vascular bundles and vascular penetrating signs, which were related to the thickening of bronchial and blood vessel edges and interstitial lesions caused by the virus (19); CT images of some COVID-19 pneumonia patients in this study showed air bronchial signs, nodular nodules, fibrosis, grids, and cord shadows may be caused by thickened interstitial cells, or accumulation of exudates (20). In general, COVID-19 pneumonia is mainly interstitial lesions, which can affect the lung parenchyma to varying degrees, and there is no cavity and other manifestations (13).

### CT Classification of Lung Lesions, Changes in Treatment Outcomes

At present, a positive nucleic acid test is the standard for the diagnosis of novel coronavirus pneumonia. Chest CT is one of the main methods for the diagnosis of pneumonitis associated with a novel coronavirus infection. Its value lies in the detection, characterization, assessment of the severity of the disease, and help clinical classification and follow-up visits after treatment. In the first diagnosis of this study, 136 patients (50.37%) showed common type diseases, which may be related to the increased secretion of T-helper-2 (Th2) cytokines (such as IL4 and IL10) that inhibited inflammation caused by coronavirus infection (15); there were 89 cases (32.96%) of severe type lesions, indicating that the disease may be in the advanced stage or the symptoms are relatively more severe than the former; 45 cases (16.67%) of critical type ill patients, there are often higher concentrations of granulocytes in these patients colony-stimulating factors, etc., suggest that increased cytokines are associated with disease severity (21). At the second examination, some of the lesions were more severe than the first, indicating that as the disease progresses, lung lesions may show a progressively worsening trend, especially in patients with underlying diseases. The interval between the two examinations is less than a week, which indicates that the inflammation of this part of the patient is in the rapid progress. It always has been found in other clinical practice that some COVID-19 patients could changed to severe with the course of the disease, and the disease progresses more rapidly (22). After more than 10 days of treatment, it gradually improved or stabilized, among the 270 patients in different types, 75 patients (27.78%) always showed the same type like before, and lesions range of 22(8.15%) patients increased, 9 patients (3.33%) death after active treatment, fortunately, 173 patients (64.08%) were recovered totally. The above results show that CT image typing and image evolution characteristics are of great significance for observing the outcome of lung lesions and guiding treatment plans, to a large extent improve the recovery rate and reduce mortality.

Limitations and deficiencies of this study: (1) This study lacks a pathological controlled study of lung damage caused by a new type of coronavirus. The CT classification is based on the speculation of imaging changes of other related coronavirus lung injuries; (2)This study lack of deeply explore the correlation between different clinical classifications and CT imaging classification of patients with COVID-19 pneumonia; (3) Some severe and critical type patients are still being hospitalized, and pulmonary CT image outcomes are still being tracked; (4) HRCT manifestations of in-patient infections of medical staff, infections of children and pregnant women were not included in this study.

To sum up, CT imaging is an important auxiliary diagnostic method, which can give early warning and assessment of lesions in lung injury. The early chest HRCT of COVID-19 pneumonia patients is mainly manifested of GGO and interstitial changes, which are obvious under the pleura; extensive GGO and infiltrates in the both lungs are typical features, and consolidation may occur in severe type. As the disease progresses, lung lesions may show a progressively worsening trend, especially in patients with underlying diseases, after more than 10 days of treatment, it gradually improved or stabilized. CT is of great significance to help clinical classification and follow-up observation after treatment.

## Data Availability Statement

All datasets generated for this study are included in the article/supplementary material.

## Ethics Statement

The studies involving human participants were reviewed and approved by Ethics Committee of Renmin Hospital of Wuhan University. Written informed consent to participate in this study was provided by the participants' legal guardian/next of kin.

## Author Contributions

XL conceived and designed the study. XL, WG, ZP, FZ, and FL performed the experiments. XL wrote the paper. WG participate in revising the paper.

## Conflict of Interest

The authors declare that the research was conducted in the absence of any commercial or financial relationships that could be construed as a potential conflict of interest.
